# A Fatty Acid Fraction Purified From Sea Buckthorn Seed Oil Has Regenerative Properties on Normal Skin Cells

**DOI:** 10.3389/fphar.2021.737571

**Published:** 2021-10-08

**Authors:** Maria Dudau, Elena Codrici, Isabela Tarcomnicu, Simona Mihai, Ionela Daniela Popescu, Lucian Albulescu, Nicoleta Constantin, Iulia Cucolea, Teodor Costache, Dan Rambu, Ana-Maria Enciu, Mihail E. Hinescu, Cristiana Tanase

**Affiliations:** ^1^ Laboratory of Biochemistry, Victor Babes National Institute of Pathology, Bucharest, Romania; ^2^ Department of Cell Biology and Histology, Carol Davila University of Medicine and Pharmacy, Bucharest, Romania; ^3^ SC Cromatec Plus SRL, Bucharest, Romania; ^4^ Department of Clinical Biochemistry, Faculty of Medicine, Titu Maiorescu University, Bucharest, Romania

**Keywords:** sea buckthorn oil, fatty acids, cell regeneration, IL-8, VEGF, keratinocytes, skin fibroblasts

## Abstract

In recent years, natural product's research gained momentum, fueled by technological advancement and open availability of research data. To date, sea buckthorn (*Hippophae rhamnoides L.* [*Elaeagnaceae*]) plant parts, especially berries, are well characterized and repeatedly tested for antioxidant activity and regenerative properties, in various cell types and tissues. However, fatty acids (FA) have been less investigated in term of biological effects, although, they are important bioactive components of the sea buckthorn fruit and oil. The aim of our work was to determine whether sea buckthorn seed oil is a suitable source of FA with regenerative properties on normal skin cells. Using high-performance liquid chromatography (HPLC) and liquid chromatography – mass spectrometry (LC-MS), we purified and characterized four fractions enriched in saturated (palmitic) and non-saturated (linoleic, alfa-linolenic, oleic) FA, which were tested for cytotoxicity, cytokine and growth factor production, and regenerative effect on normal keratinocytes and skin fibroblasts. Evidence is presented that the palmitic acid enriched fraction was a suitable sea buckthorn seed oil derived product with cell proliferation properties on both skin cell types.

## Introduction

Accelerated development of biotechnologies during the last decades allowed thorough biochemical investigation of medicinal plants, such as sea buckthorn (*Hippophae rhamnoides L.* [*Elaeagnaceae*]. One of the final products of sea buckthorn is the oil that can be extracted from the whole berry or seeds. Depending on its origin and method of extraction (Kumar et al., 2011; Gong et al., 2015) and also on its genetic background (Li et al., 2020), sea buckthorn oil may show variations in biochemical composition (Dulf, 2012; Pariyani et al., 2020) but is unanimously appreciated for its antioxidant (Ivanišová et al., 2020) and anti-inflammatory properties (Balkrishna et al., 2019; Baek et al., 2020; Pundir et al., 2021). The constancy of reports of beneficial properties of sea buckthorn oil in *in vitro* (Gęgotek et al., 2018) and *in vivo* models (Wang et al., 2016; Bouras et al., 2017; Czaplicki et al., 2017) (reviewed in Solà Marsiñach and Cuenca, 2019) prompted a detailed analysis of those compounds with bioactive properties, now known to be phenolics, flavonoids, and carotenoids, as well as lipid soluble vitamins and their precursors (Guo et al., 2017; Criste et al., 2020) (reviewed in Zielińska and Nowak, 2017).

Although they are important components of the sea buckthorn, fatty acids (FA) have been less investigated in terms of biological effects (for a recent review on their impact on human health, see (Solà Marsiñach and Cuenca, 2019). It is known that sea buckthorn oil is rich in saturated and unsaturated FA and has an unusually high concentration of palmitoleic acid with proven benefits on skin health (Weimann et al., 2018; Niimi et al., 2021). Little is known about the implication of other FAs in cellular regeneration, given the fact that they are a major component of cell membrane lipids. The aim of our work was to determine whether sea buckthorn seed oil is a suitable source of FA with potential regenerative properties on normal skin cells and whether these exploitation products are a match for commercially available analytical standards.

## Materials and Methods

### FA-Enriched Fraction Production

Four fractions enriched in FA were obtained from sea buckthorn seed oil by preparative liquid chromatography (LC). A system consisting of a PerkinElmer (Waltham, MA, United States) Flexar FX-15 quaternary pump connected to a photodiode array detector equipped with deuterium and tungsten lamps (wavelength range 190–700 nm) was used. For this study, sea buckthorn oil was injected manually via a 400-µL loop, after a simple dilution step with ethanol to a concentration of 100 ng/ml. FA separation was achieved on a Cosmosil 5C18-AR-II column (150 mm × 20 mm), produced by Nacalai Tesque (Kyoto, Japan), eluted at 6 ml/min with a mobile phase composed of acetonitrile/water (95/5, v/v, isocratic mode). The detector was set at 208 mm. LC solvents were purchased from Merck (Darmstadt, Germany), and ultrapure water was obtained with a MilliQ system from Millipore Merck (Molsheim, France). The standards of FA were bought from Sigma-Aldrich (St. Louis, MI, United States). A Gilson fraction collector model FC 203B (Middleton, WI, United States) was placed in the system after the chromatographic column, and the eluent corresponding to the specific retention times of the selected FA was collected in 6-ml polypropylene tubes. Stearic acid, accounting for less than 1% of the oil composition, was not collected. The eluate from 20 injections for each fraction was reunited in 50-ml polypropylene tubes and evaporated up to a volume of 1 ml, from which 100 µL were used for chemical characterization, and the rest for *in vitro* testing. The FA concentrations in each fraction were measured by LC coupled with mass spectrometry (MS), with an in-house developed method previously described (Dudau et al., 2021). The obtained fractions were diluted with methanol in order to fit in the range of the calibration curves prepared with dilutions of the FA standards, analyzed in the same run with the respective calibration curves, and then quantified.

### Cell Culture and Cell Treatments

Normal human keratinocytes (NHEK – Lonza) and normal human dermal fibroblasts (HDFa -ATCC) were maintained in standard cell culture conditions, using the cell medium recommended by the producer (Dermal Cell Basal Medium, PCS-200-030 ATCC, supplemented with Keratinocyte Growth Kit PCS-200-040, and Fibroblast Basal Medium, PCS-201-030 supplemented with Fibroblast Growth Kit PCS-201-041, respectively). Purified fractions and analytical standards of FA were prepared as stock solution in ethanol 100% and stored at −20°C until the day of use. Tested dilutions were prepared fresh for each experiment. Lipopolysaccharide (LPS – L4391, Sigma) was stored as a stock solution of 1 mg/ml in Hank’s balanced salt solution at −20°C until the day of use and reconstituted with complete cell medium in working solution (1 µg/ml).

### Cell Toxicity and Proliferation Assays

Cytotoxicity was assessed by cellular lactate dehydrogenase (LDH) release (CytoTox 96^®^ Non-Radioactive Cytotoxicity Assay, Promega) (Allen and Rushton, 1994). 10,000 cells were seeded in triplicates overnight in 96-well plates and incubated the next day with serial dilutions of the stock solution for additional 72 h. Two controls were included: a negative control (cell culture medium supplemented with vehicle) and a positive control (addition of Cell Lysis Reagent 10x, Promega, 45 min prior to LDH detection). Additional triplicates of cell-free wells were incubated with test solutions, for background subtraction. For LDH analysis, 50 µl of supernatant was collected from each well and incubated with the assay reagent for 30 min in the dark. After the addition of the stop solution, absorbance was read at 490 nm by using a microplate reader Anthos Zenyth 3100 (Austria). Cytotoxicity was assessed as % out of the positive control, according to the formula % cytotoxicity = 100 × (Sample OD−background OD)/(average OD of positive control – average OD of background).

Proliferation was assessed by MTS [3-(4,5-dimethylthiazol-2-yl)-5-(3-carboxymethoxyphenyl)-2-(4-sulfophenyl)-2H-tetrazolium] assay (CellTiter 96^®^ AQueous One Solution Reagent, Promega) (Cory et al., 1991). 10,000 cells were seeded in triplicates o/n in 96-well plates and incubated the next day with cell culture medium and/or serial dilutions of stock solution for 72 h. A positive control (non-treated cells) was included. Additional triplicates of cell-free wells were incubated with test solutions for background subtraction. On the day of the reading, the cell medium was removed, and 100 µl of fresh medium and 20 µl of MTS reagent were added to each well. The plate was incubated at 37°C for 3 h in a humidified 5% CO2 atmosphere. Absorbance was read at 490 nm by using a microplate reader Anthos Zenyth 3100 (Austria). Proliferation was assessed as % of the control: % of proliferation = 100 × (sample OD−background OD)/(average OD of control—average control OD of background).

### Oil Red Staining

Cells were fixed in 3.7% formaldehyde for 30 min to 1 h, washed with water, and then incubated with 60% isopropanol for 5 min. Oil Red O Stock solution (MAK 194 Sigma-Aldrich) was reconstituted with 100% isopropanol. Oil Red O working solution was prepared, 15 min before staining, by mixing three parts of Oil Red O stock solution and two parts water and then filtered through a Whatman No.1 filter paper. The cells were stained with Oil Red O working solution for 10 min at room temperature and after that washed 2–5 times with water until no excess stain was seen (for further details regarding protocol, address the product technical bulletin). 4′,6-diamidino-2-phenylindole (DAPI) staining can be performed after this step for 10 min, at room temperature, and protected from light. The red lipid droplets were visualized by microscopy.

### Scratch-Wound Assay

Cells were seeded in a 24-well plate to full confluence and then a scratch was performed with a 200-µL tip. Image capture of the scratch was taken at different time points (2, 4, 6, 18, 36, and 48 h) with a phase-contrast Evos microscope. Measurement of the nude area was performed with ImageJ software (Suarez-Arnedo et al., 2020).

### Real-Time Impedance Readings

Cell proliferation was assessed using RTCA DP platform (Agilent Technologies), as previously reported (Chirică et al., 2021). Cells were seeded in E-16 plates, at a density of 20,000 cells/well, and let to adhere for 20 h in complete cell medium. After 20 h, the cells were treated with purified fractions and FA analytical standards at 25 µM. The cell index was normalized to 1 before addition of supplements, to discard any seeding differences or proliferation differences between wells. Readings were collected every 15 min until cells reached a plateau. Using xCElligence software, the doubling time of treated cells was calculated, over a period of 40 h, during which the cell index was on an ascending trend.

### Multiplexing Assay

Cell culture supernatants were obtained as mentioned earlier and then a Multiplex Magnetic Luminex Assay Human Premixed Multi-Analyte analysis (R&D Systems, Minneapolis, MN, United States) was performed to assess the level of 7 different biomarkers [interleukin (IL)-1beta, IL-6, IL-8, IL-10, IL-12p70, tumor necrosis factor alpha (TNFα), and vascular endothelial growth factor (VEGF)-A], as previously described (Ionescu et al., 2018). In brief, the magnetic beads were incubated with buffer, cytokine standards, or samples, according to the manufacturer’s protocols. All further incubations with biotin–antibody cocktail and streptavidin–phycoerythrin were performed at room temperature, in the dark with shaking at 800 ± 50 rpm. Multiplex data acquisition was achieved using Luminex®200™ platform (Luminex Corp, Austin, TX 78727, United States), and analysis was performed using xPONENT 4.2 software; the calibration curves were generated with a 5-parameter logistic fit. Duplicate samples were used for all the specimens, and the average concentrations were used for statistical analysis.

### ELISA

IL-8 (cat no. 431507), IL-6 (cat no. 430507), and TNFα (cat no. 430207) were measured using commercial Legend Max quantitative assays (BioLegend, San Diego, CA, United States). Standards and samples—cell culture supernatants (50 μL)—were added to the plates and incubated at room temperature for 2 h, with shaking at 200 rpm. All further incubations with detection antibody (100 μL), avidin–horseradish peroxidase (HRP) solution (100 μL), substrate solution (100 μL), and stop solutions (100 μL) were performed according to the manufacturer’s protocols. The samples were analyzed in duplicate, and the absorbance was read at 450 and 570 nm, using a Sunrise-Basic Tecan Microplate Reader (Tecan Group Ltd., Männedorf, Switzerland). The absorbance at 570 nm was subtracted from the absorbance at 450 nm. The data were calculated with computer-based curve-fitting software (Magellan) using a 5-parameter logistics curve-fitting algorithm. IL-8, IL-6, and TNFα were expressed in picograms per milliliter of the sample.

### Statistical Data Analysis

Statistical data analysis was performed with GraphPad v7 by one-way ANOVA and Dunnett’s multiple comparison, where data were compared to the control. *p < 0.05, **p < 0.01, ***p < 0.001, ****p < 0.0001).

## Results

### Four Fractions (F1-F4) Were Purified From the Sea Buckthorn Seed Oil

**FIGURE 1:**
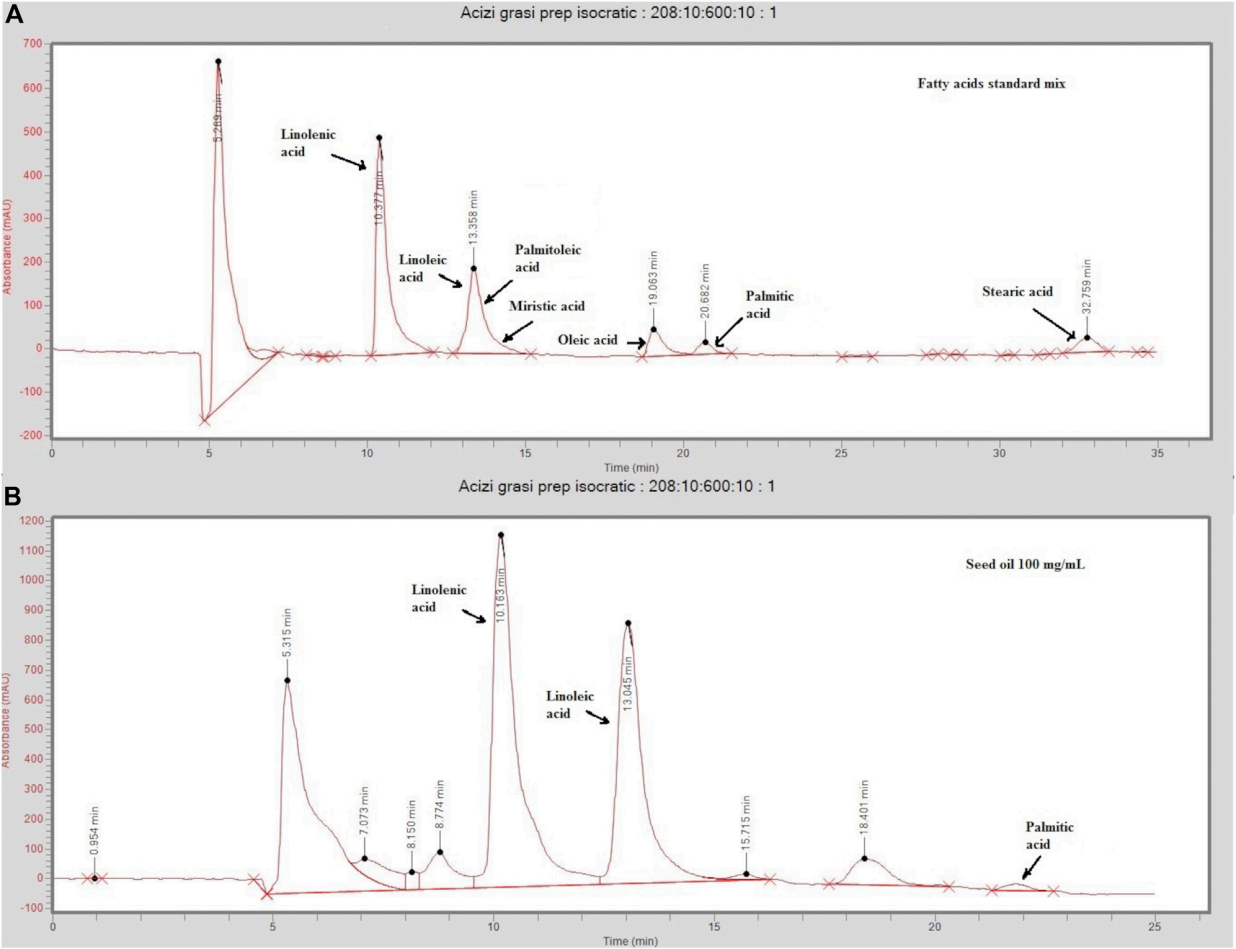
Chromatograms of a standard mix solution **(A)** and sea-buckthorn seed oil **(B)** injected on the preparative (Cosmosil 5C18-AR-II 150 mm × 20 mm) eluted at 6/ml/min in isocratic conditions (acetonitrile/water 95:5, v/v). The standard mix contained linolenic and linoleic acids at 50 µg/ml; oleic and palmitoleic acids at 100 µg/ml; and myristic, palmitic, and stearic acids at 300 µg/ml in ethanol. Injected sea buckthorn seed oil was diluted at 100 ng/ml in ethanol.

In the conditions selected for preparative chromatography, linolenic acid eluted at 10.1 min, oleic, palmitic, and stearic acids eluted at 18.7, 21.7, and 33 min, respectively, while linoleic and palmitoleic acids were not separated, eluting together at 13.1 min (Figure 1). A standard mix containing linolenic and linoleic acids at 50 µg/ml; oleic and palmitoleic acids at 100 µg/ml; and myristic, palmitic, and stearic acids at 300 µg/ml in ethanol was used for separation optimization*.*

Four FA-enriched fractions were collected. Fraction 1 contains linolenic acid (ALA), fraction 2 contains linoleic (LA) and palmitoleic acid (POA), fraction 3 contains oleic acid (OA), and fraction 4 contains palmitic acid (PA). A gradient method aimed at separating LA and POA was tested, but it resulted in high retention times and too much solvent consumption. Thus, as a compromise, isocratic conditions were selected, and LA and POA were collected in the same fraction. The concentrations are summarized in Table 1. Representative chromatograms of the four fractions analyzed by LC/MS are presented in Supplementary Figures S1–S4.

**TABLE 1:** Fatty acid content of the four enriched fractions.

Enriched fraction	F1	F2	F3	F4
Compound				
Linolenic acid	2.768 mg/ml			
Linoleic acid		4.12 mg/ml		
Oleic acid			11.796 mg/ml	
Palmitoleic acid		2.228 mg/ml		
Palmitic acid				7.944 mg/ml

### From the Four Purified Fractions, Only Two (F1 and F4) Are Non-Toxic

**FIGURE 2:**
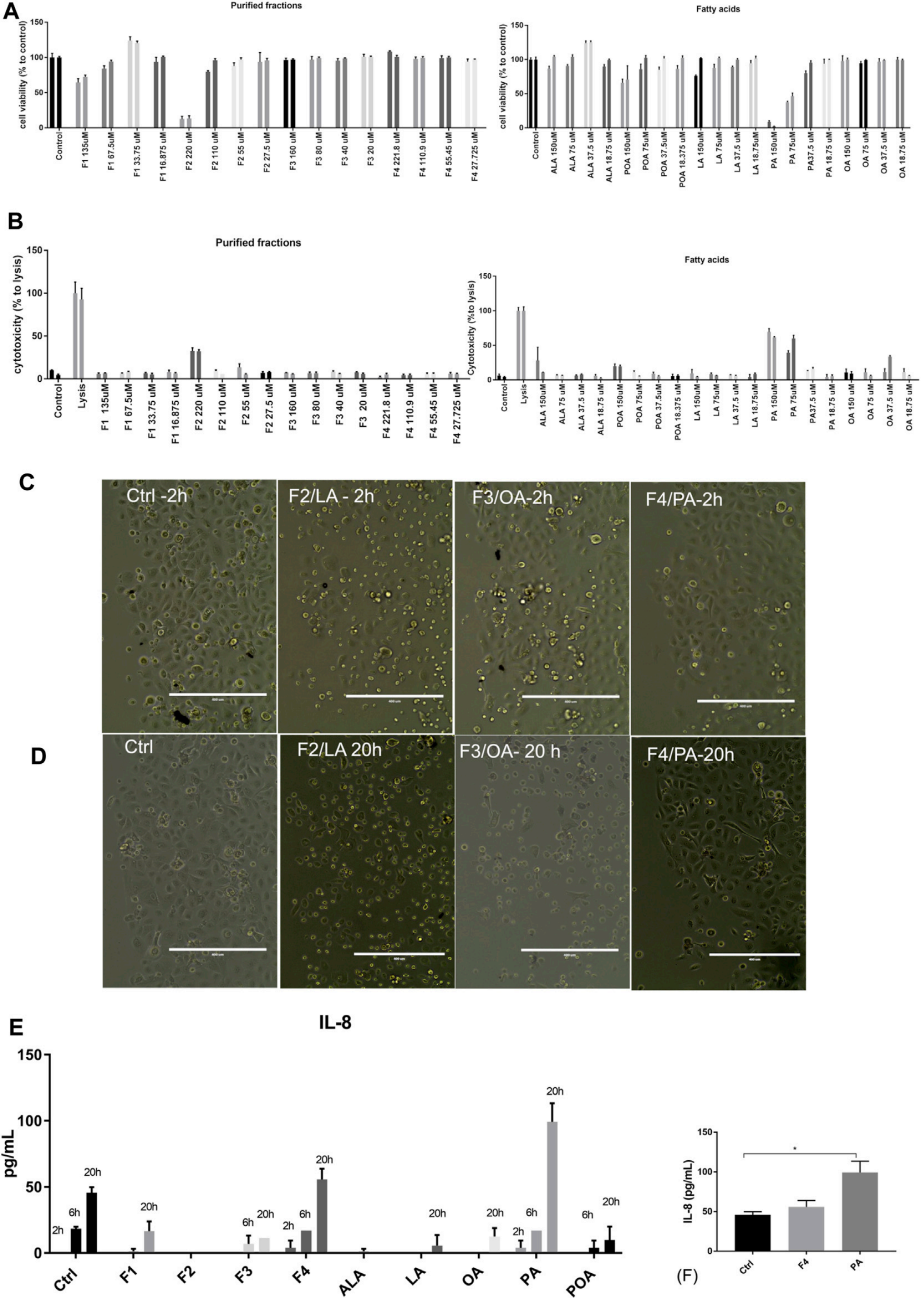
Viability and cytotoxicity of purified fractions on normal human keratinocytes. Viability of cells treated with purified fractions and analytical standards was assessed by MTS **(A)** and LDH release **(B)**. Cells were incubated for 24 h (first bar of the sample) and 48 h (second bar of the respective sample) with the indicated concentrations. Bars represent the average of triplicates±S.D. Cell morphology of cells treated with 25 µM purified fractions (F1–F4) was assessed by phase-contrast microscopy at 2 h **(C)** and 20 h **(D)**. The name of the fraction in this figure also indicates the FA enriched in the respective fraction (e.g., F4/PA = fraction 4, enriched in PA). Supernatant of cells treated with 25 µM purified fractions and corresponding FA analytical standards were tested for cytokine release by multiplexing **(E)** and confirmed by ELISA **(F)**.

The four purified fractions were tested for cytotoxicity and putative inflammatory effect on normal human keratinocytes, and cross-referenced with analytical standards of FA. They were investigated in terms of cytotoxicity and inducement of pro- and anti-inflammatory cytokines in cell culture. Biochemical analysis was complemented by morphology analysis of the treated cell cultures. Cell viability analysis by MTS showed that purified fractions were not cytotoxic, except for high concentration of F2, enriched in LA. When compared to analytical standards, this effect was not duplicated but only PA impaired cell proliferation in a dose-dependent manner, which was not observable in the respective purified fraction (F4). Both analytical standard ALA and purified fraction F1 enriched in ALA showed a proliferative effect on normal keratinocytes at low concentrations, but F1 showed dose-dependency (Figure 2A). These data were correlated with LDH release (Figure 2B). Future experiments were performed at 25 µM for both purified fractions and FA analytical standards.

However, morphological inspection of cell cultures showed that even at low concentrations F2 induced detachment of cells or change of morphology from flattened form to round after 2 h of incubation (Figure 2C); this effect was observed after 20 h of incubation for F3, enriched in OA (Figure 2D).

We also investigated by multiplexing assay and ELISA that the release in the cell medium of pro-inflammatory (IL-6, IL-8, and TNFα) and anti-inflammatory cytokines (IL-4 and IL-10), at three different times. The only cytokine that was significantly expressed and modulated over time was IL-8 (Figures 2E,F). As compared to PA, F4 showed a protective effect, lowering IL-8 at levels close to the control. Based on the data obtained so far, we selected for further testing F1 and F4 at 25 µM.

### The Two Selected Fractions (F1 and F4) Are Uptaken by Keratinocytes and Do Not Induce Inflammation

**FIGURE 3:**
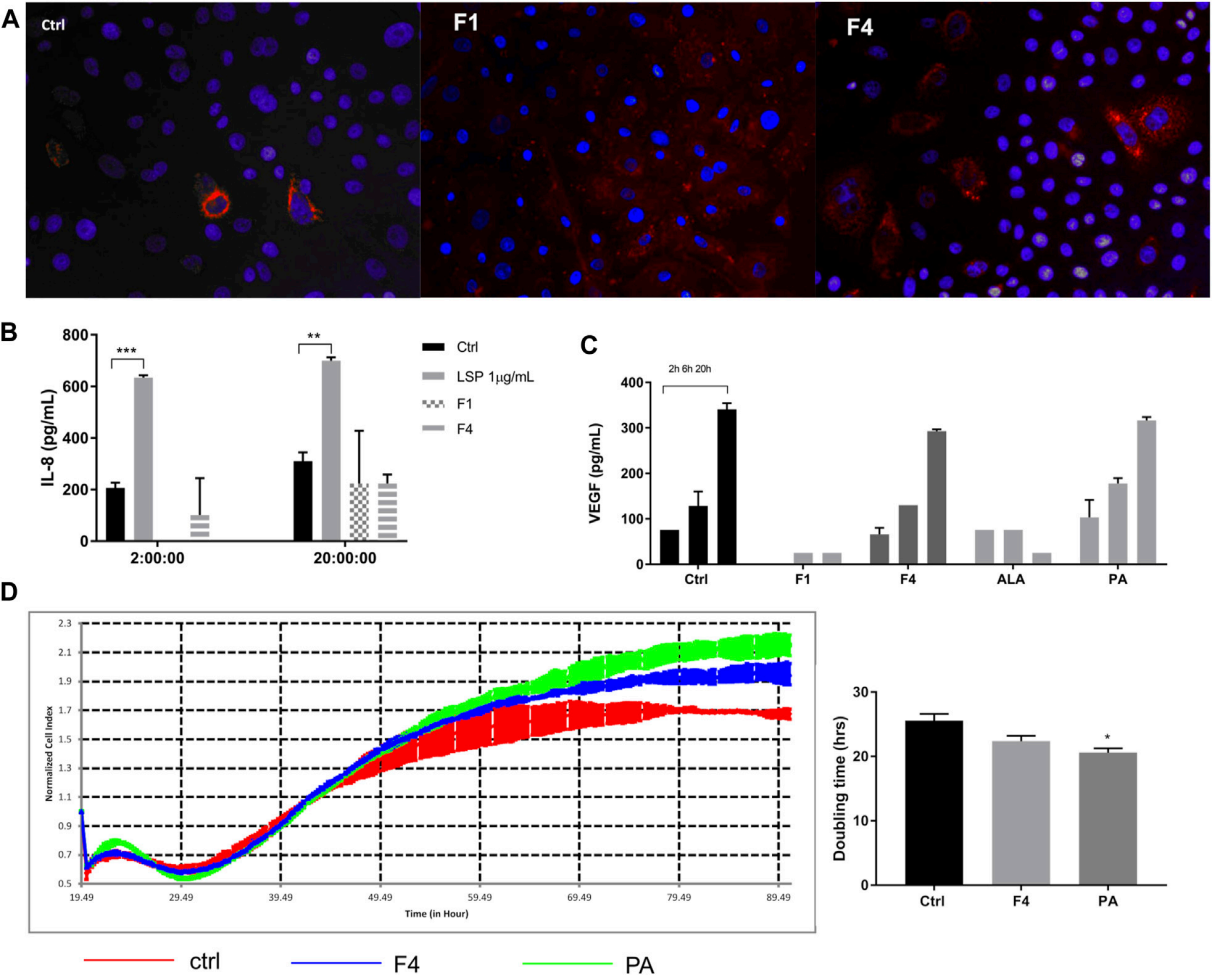
Investigation of selected purified fraction effect on human normal keratinocytes. Assessment of ALA- and PA-enriched fractions uptake of normal keratinocytes by Oil Red showed good cellular penetrability **(A)**. Treatment with purified fractions did not induce an inflammatory response, as assessed by ELISA for IL-8 **(B)**. VEGF expression, assessed by multiplexing, showed that F4 and its corresponding FA analytical standard (PA) followed the same trend as the control **(C)**. Long-time monitoring of the cell index using real-time impedance reading combined with doubling time analysis showed that PA significantly stimulates cell proliferation of normal human keratinocytes **(D)**.

In order to see whether the purified fractions are not only nontoxic but also biocompatible, we tested whether the selected fractions are uptaken by the cell and if the lipid uptake induces an inflammatory response compared to LPS stimulation. We noticed that in 24 h, more cells have lipid droplets if treated with F1 and F4 (Figure 3A); therefore, the FA from purified fractions are able to penetrate the cell membrane. As noticed previously, normal keratinocytes secrete IL-8 in the extracellular medium in normal conditions, as well as under F4 and PA treatment. F1 treatment only induces IL-8 secretion on long term at levels comparable to F4. However, these levels are significantly lower than those induced by LPS stimulation (Figure 3B), which supports the idea that the purified fractions do not induce acute inflammation by themselves. Finally, we also noticed that in F4- and PA-treated cells, VEGF release in the cell medium was similar to the control, following the same time-dependent profile (Figure 3C). This effect was not observed for neither F1 nor ALA. However, in terms of growth factor stimulation, F1 failed to induce a sustained VEGF expression, as opposed to F4. Therefore, we selected F4 as a possible candidate for skin cell regeneration and confirmed its pro-regenerative abilities by real-time impedance readings. The xCelligence readings showed that during the first 24 h, there was no significant difference between treated and non-treated cells. The cellular indexes begin to diverge around 30 h, when the control started to flatten the proliferation slope and reached a plateau sooner than treated cells (Figure 3D). Doubling time analysis showed that treatment with 25 μMPA significantly stimulates cell proliferation. The same trend was observed for the respective purified fraction, but the difference did not reach statistical significance.

### Palmitic Acid and Corresponding Purified Fraction (F4) From Sea Buckthorn Seed Oil also Favor Cell Proliferation of Normal Skin Fibroblasts

**FIGURE 4:**
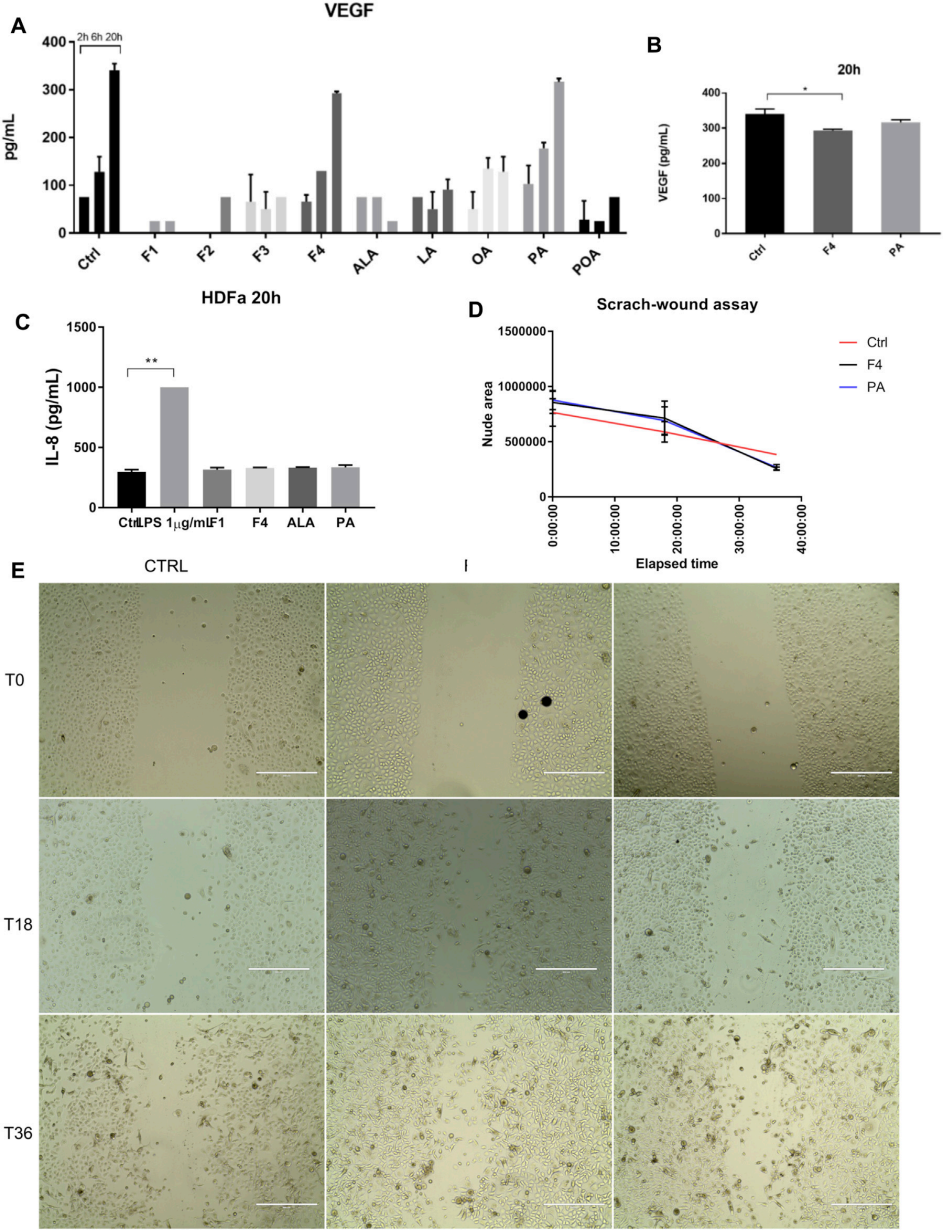
Investigation of selected purified fraction effect on human normal fibroblasts. VEGF expression showed a similar trend in F4 and PA treated cells, when assessed by multiplexing **(A)** and confirmed by ELISA **(B)**. IL-8 expression was not altered by cell treatment, as shown by ELISA analysis **(C)**. LPS was used as positive control of inflammation. Bars represent the average of duplicates ±S.D., and each treatment was assessed at three different time points 2, 6, 20 h (*p < 0.05, **p < 0.01, ****p < 0.0001, One-way ANOVA, Dunnett’s multiple comparison). Cell migration was stimulated after 20 h **(D)**, as assessed by scratch-wound assay **(E)**. Phase-contrast, 10x. Scale bar 400 µm.

Because healthy skin contains both epidermal and connective tissue cells, we also tested the effect of selected fraction on proliferation and migration of normal skin fibroblasts. We also determined cytokine and VEGF release in the cell medium, following the treatment of fibroblasts with all purified fractions and FA analytical standards previously tested for normal keratinocytes (Figure 4A), by multiplexing and confirmed by ELISA (Figure 4B). The analysis of VEGF showed that it is produced in a linear trend, similar to control in both F4- and PA-based cell treatments. However, ELISA validation of VEGF secretion showed that F4-treated cells have a lower expression than that of the control. No other tested cytokine increased by the treatments applied. The lack of response was confirmed by ELISA for IL-8, where the fractions of interest were compared to 1 µM LPS stimulation (Figure 4C). In order to assess whether lower VEGF secretion impairs on cell migration of fibroblasts, we performed a scratch-wound assay (Figure 4E). Time-lapse analysis showed that during the first 18–20 h, the evolution of non-treated and treated cells is quite similar (Figure 4D). However, after this interval, treated cells accelerated their proliferation, covering a wider surface than control cells, which had a linear trend of evolution. Both F4- and PA-treated cells behave in a similar manner.

## Discussion

Sea buckthorn seed oil has many benefits reported on skin health, such as wound healing (Upadhyay et al., 2009; Edraki et al., 2014; Ito et al., 2014; Kaur and Kapoor, 2018) modification of sebum characteristics, and improvement of atopic skin (Yang et al., 2000; Weimann et al., 2018; Yao et al., 2021). Some of these benefits are attributed to POA, an unsaturated FA, enriched in sea buckthorn pulp/peel and absent in seeds (Yang and Kallio, 2001; Fatima et al., 2012) (benefits of POA on skin health are reviewed in Solà Marsiñach and Cuenca, 2019). However, other FA found in sea buckthorn oil may also contribute to skin health. For example, PA, found in sea buckthorn seeds, is known to be abundant in normal human skin; it contributes to an efficient lipid barrier in *in vitro* models of skin (Mieremet et al., 2019) and is used by cells as a precursor for longer chain saturated FA. It is being used in skin cosmetic products and considered safe for topic administration. However, as a saturated FA, it is usually overlooked as most studies focus on unsaturated FA (omega 3, 6). Also, composition in FA of various sea buckthorn derived products varies with area, season of harvesting, and even from cultivar to cultivar (Yang and Kallio, 2002; St. George and Cenkowski, 2007; Vuorinen et al., 2015; Criste et al., 2020). Regardless of these variations, we aimed to purify sea buckthorn seed oil from various fractions enriched in saturated and unsaturated FA and test their effect on skin cell proliferation. We separated four fractions, enriched in ALA, LA, OA, and PA, compatible with cellular viability in low micromolar range (50 µM or less) and physiologically uptaken by cells. Our study demonstrated that other fractions than POA can be used to develop skincare-related products, despite variations in chemical compositions of the starting product. Of the four purified fractions, ALA and PA – enriched fractions did not alter cellular morphology of normal keratinocytes and did not induce inflammation in normal keratinocytes and dermal fibroblasts (assessed by release in the cell medium of the pro-inflammatory cytokines—IL-6 and TNFα). Production of IL-8 was noted in both cell types at levels similar to control. It is known that keratinocytes are a rich source of IL-8, which stimulates their migration, and it is thought to induce wound healing (Jiang et al., 2012).

Another cytokine associated with cell proliferation is VEGF, also produced by keratinocytes, which acts via autocrine signaling (Benhadou et al., 2019) and stimulates keratinocyte proliferation (Bae et al., 2015). It is to be noted that overexpression of VEGF is associated with psoriasis (Gerkowicz et al., 2018) and malignancy (Lichtenberger et al., 2010), therefore a balanced expression is to be achieved following application of a treatment. Our PA-enriched fraction lowered the expression of VEGF, therefore it does not raise concern issues in this matter.

Finally, of the two fractions, PA-enriched fraction supported keratinocytes and fibroblast proliferation, possibly by additionally fueling the cellular metabolism. This effect became apparent around 30 h of incubation and became significant after 48 h.

In conclusion, we successfully purified several FA-enriched fractions from sea buckthorn seed oil, out of which the PA-enriched fraction supported cell proliferation for both keratinocytes and dermal fibroblasts, without triggering inflammation or excessive VEGF synthesis. This fraction is suitable for a topic formulation intended for skin application, for further *in vivo* testing. Despite the absence of POA in the sea buckthorn seed oil, we demonstrated that other fractions can be used to develop skincare-related products.

## Data Availability

The original contributions presented in the study are included in the article/Supplementary Material; further inquiries can be directed to the corresponding author.
