# Supplementary figures and images for: A Fatty Acid Fraction Purified From Sea Buckthorn Seed Oil Has Regenerative Properties on Normal Skin Cells
Source: Front Pharmacol. 2021 Oct 8;12:737571. doi: 10.3389/fphar.2021.737571 (PMC8547141; doi:10.3389/fphar.2021.737571)

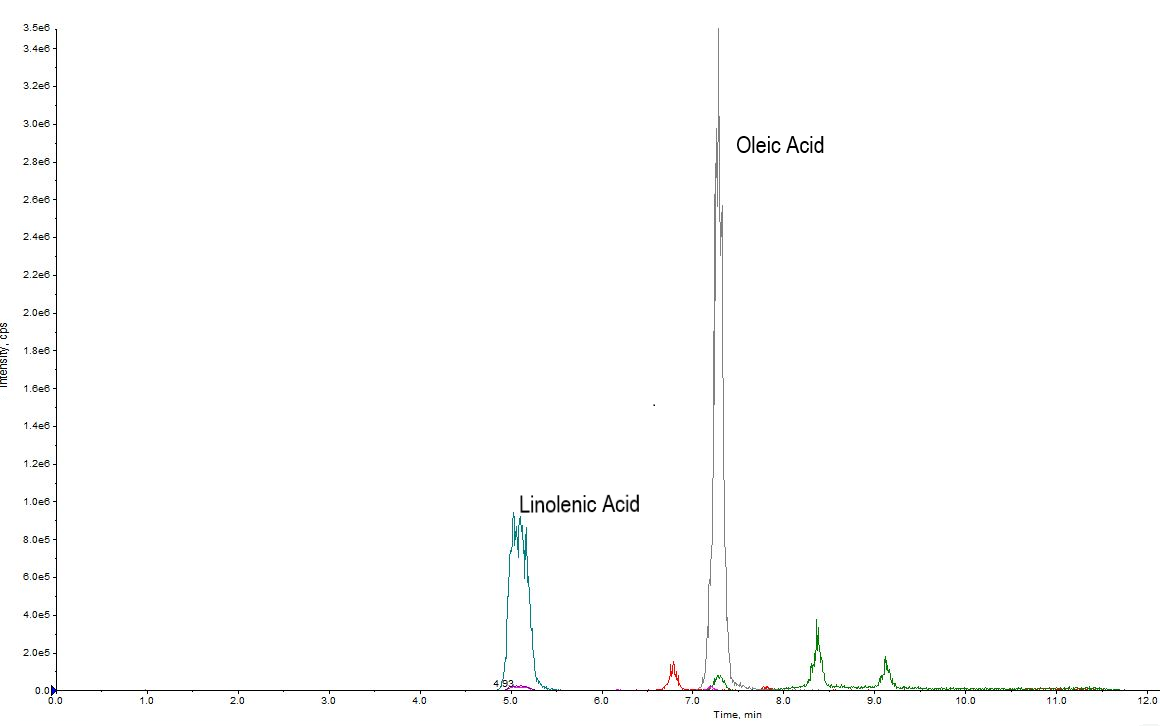

Supplement: Supplementary file 1 [file DataSheet1.ZIP › Figure S1 bmp.bmp]

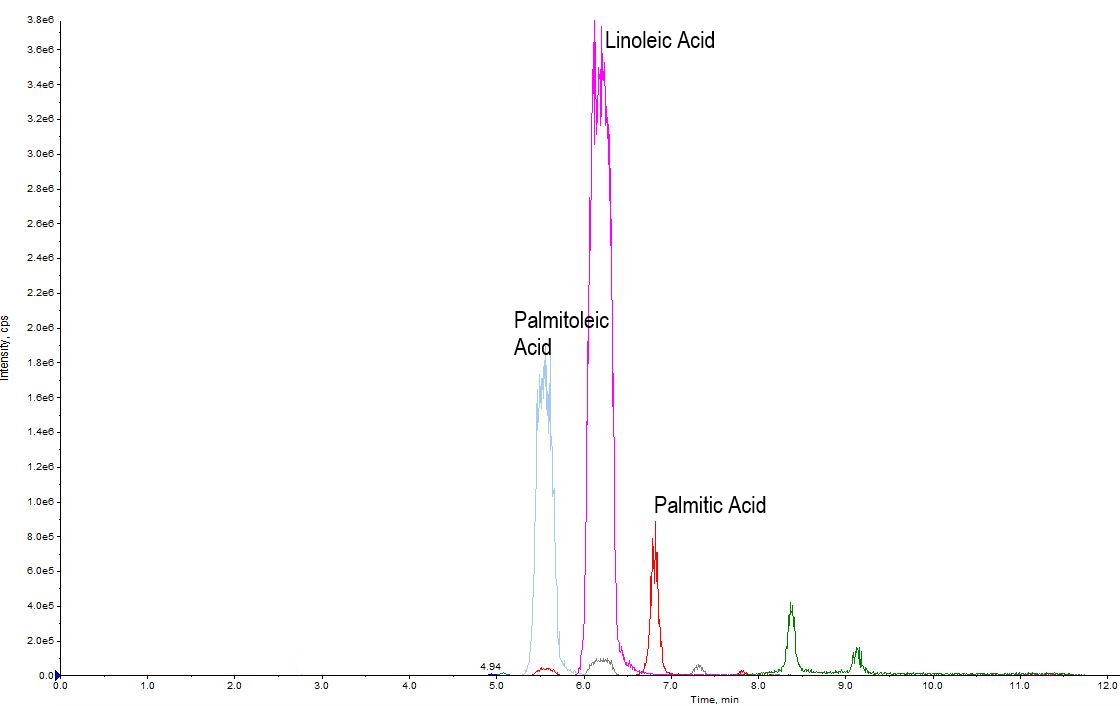

Supplement: Supplementary file 1 [file DataSheet1.ZIP › Figure S2.bmp]

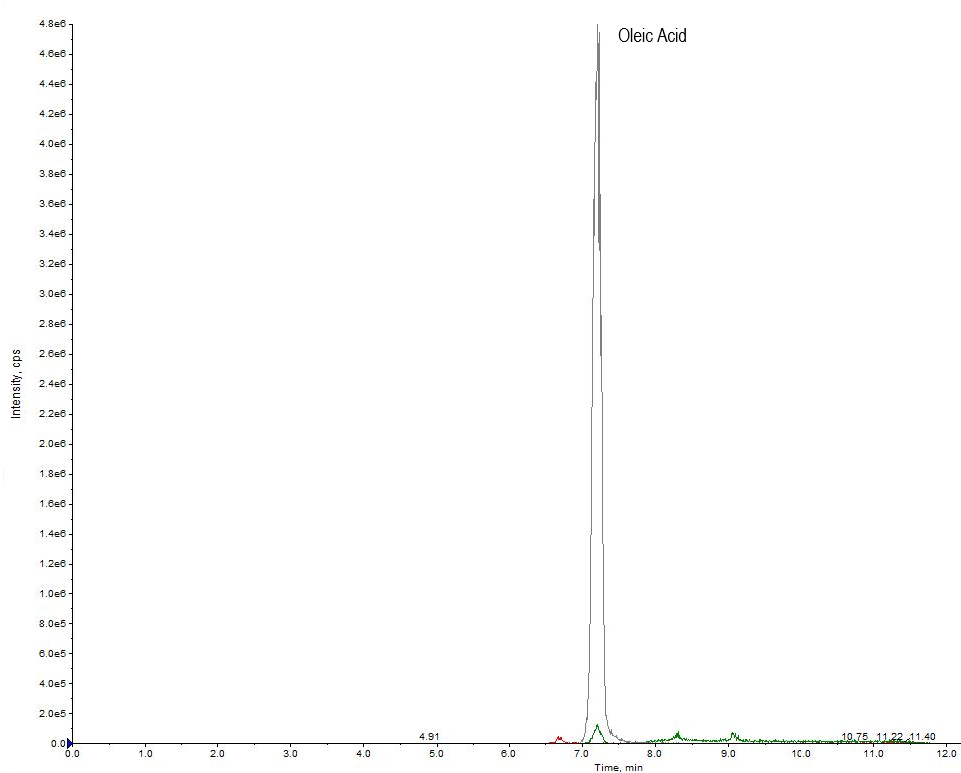

Supplement: Supplementary file 1 [file DataSheet1.ZIP › Figure S3.bmp]

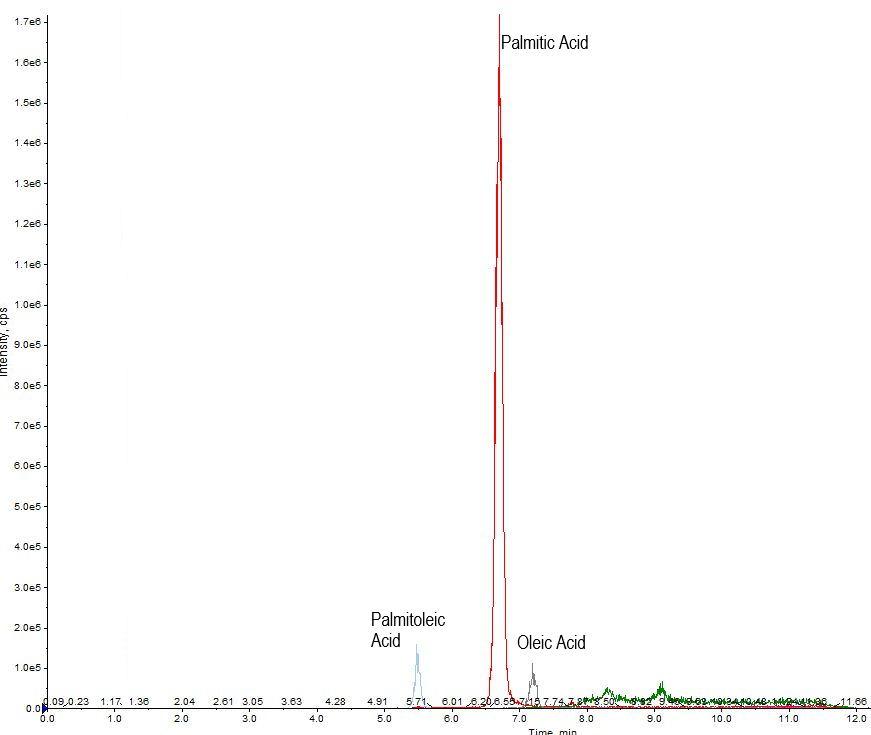

Supplement: Supplementary file 1 [file DataSheet1.ZIP › Figure S4.bmp]
